# SNP Based Trait Characterization Detects Genetically Important and Stable Multiple Stress Tolerance Rice Genotypes in Salt-Stress Environments

**DOI:** 10.3390/plants11091150

**Published:** 2022-04-24

**Authors:** Sanjoy K. Debsharma, Mohammad Akhlasur Rahman, Mohammad Ruhul Quddus, Hasina Khatun, Ribed F. Disha, Popy R. Roy, Sharif Ahmed, Mohamed El-Sharnouby, Khandakar Md. Iftekharuddaula, Salman Aloufi, Fahad M. Alzuaibr, Mohammed Alqurashi, Mohamed I. Sakran, Mohammad Shahjahan Kabir

**Affiliations:** 1 Bangladesh Rice Research Institute (BRRI), Gazipur 1701, Bangladesh; sanjoybrri@gmail.com (S.K.D.); rquddus265@gmail.com (M.R.Q.); hasinabrri09@gmail.com (H.K.); ribedbau@gmail.com (R.F.D.); kiftekhar03@yahoo.com (K.M.I.); kabir.stat@gmail.com (M.S.K.); 2 Seed Certification Agency, Ministry of Agriculture, The Peoples Republic of Bangladesh Government, Gazipur 1701, Bangladesh; popyroy31@gmail.com; 3 International Rice Research Institute, Bangladesh Office, Dhaka 1213, Bangladesh; s.ahmed@irri.org; 4 Department of Biotechnology, College of Science, Taif University, P.O. Box 11099, Taif 21944, Saudi Arabia; m.sharnouby@tu.edu.sa; 5 Department of Biotechnology, Faculty of Science, Taif University, Taif 21974, Saudi Arabia; s.aloufi@tu.edu.sa (S.A.); m.khader@tu.edu.sa (M.A.); 6 Department of Biology, Faculty of Science, University of Tabuk, Tabuk 71491, Saudi Arabia; falzuaiber@ut.edu.sa; 7 Biochemistry Section, Chemistry Department, Faculty of Science, Tanta University, Tanta 31527, Egypt; msakran@ut.edu.sa; 8 Biochemistry Department, Faculty of Science, University of Tabuk, Tabuk 47512, Saudi Arabia

**Keywords:** salinity stress, advanced breeding lines, stable yield, trait genotyping, multiple stress, *Oryza sativa*

## Abstract

Soil salinity is a major constraint to rice production in coastal areas around the globe, and modern high-yielding rice cultivars are more sensitive to high salt stress, which limits rice productivity. Traditional breeding programs find it challenging to develop stable salt-tolerant rice cultivars with other stress-tolerant for the saline environment in Bangladesh due to large yield variations caused by excessive salinity fluctuations during the dry (*boro*) season. We examined trait characterization of 18 advanced breeding lines using SNP genotyping and among them, we found line G6 (BR9621-B-1-2-11) (single breeding line with multiple-stress-tolerant QTL/genes) possessed 9 useful QTLs/genes, and two lines (G4:BR9620-2-7-1-1 and G14: IR 103854-8-3-AJY1) carried 7 QTLs/genes that control the desirable traits. To evaluate yield efficiency and stability of 18 rice breeding lines, two years of field experiment data were analyzed using AMMI (additive main effect and multiplicative interaction) and GGE (Genotype, Genotype Environment) biplot analysis. The AMMI analysis of variance demonstrated significant genotype, environment, and their interaction, accounting for 14.48%, 62.38%, and 19.70% of the total variation, respectively, and revealed that among the genotypes G1, G13, G14, G17, and G18 were shown to some extent promising. Genotype G13 (IR 104002-CMU 28-CMU 1-CMU 3) was the most stable yield based on the AMMI stability value. The GGE biplot analysis indicates 76% of the total variation (PC1 48.5% and PC2 27.5%) which is performed for revealing genotype × environment interactions. In the GGE biplot analysis, genotypes were checked thoroughly in two mega-environments (ME). Genotype G14 (IR103854-8-3-AJY1) was the winning genotype in ME I, whereas G1 (BR9627-1-3-1-10) in ME II. Because of the salinity and stability factors, as well as the highest averages of grain yield, the GGE and AMMI biplot model can explain that G1 and G13 are the best genotypes. These (G1, G6, G13, G14, G17, and G18) improved multiple-stress-tolerant breeding lines with stable grain yield could be included in the variety release system in Bangladesh and be used as elite donor parents for the future breeding program as well as for commercial purposes with sustainable production.

## 1. Introduction

Rice (*Oryza sativa* L.) is the world’s most widely grown crop, which delivers about 75% of the essential calories and 55% of the protein intake in people’s daily diet in Bangladesh [1]. The salinity-affected areas of Bangladesh cover around 2.85 million hectares of land in the southern coastal zone [2] which supplies 16% of the country’s total rice production [3]. This coastal belt is increasingly meeting salt invasions that hamper rice production and other growing crops [4].

Characterizing genotypes using trait-specific SNPs is important to investigate the frequency of favorable alleles for different traits of interest related to biotic, abiotic stresses, and grain quality characters. The high throughput SNP platform has created this opportunity to select genotypes using modern breeding approaches such as marker-assisted forward breeding and genomic selection for selecting elite parents with superior qualities. The availability of several useful QTLs/genes in a breeding program indicates that the program could meet the multifarious demand of stakeholders from different social segments by enhancing the genetic gain.

Adaptation of varieties to the environment and location-specific variations is essential for the stabilization and maximization of crop outputs over both years and environments. Sometimes, its production is directly or indirectly impacted by difficult conditions such as sudden havoc rain, long-time swells, heavy or uneven rainfall, and cyclones that rain out the saltwater in the local cultivated areas. The salinity stress adversely affects the land area. Both the salt-affected areas and the human population is increasing gradually and steadily. To feed and nourish the quickly evolving population of Bangladesh, salinity disturbed regions should be brought under paddy cultivation to sustain food security in the country, as rice is the basic food [5,6,7,8,9]. Multi-environmental yield trial for various crops around the world [10,11] is neither alone used to recognize high yielding, but further to analyze sites representing the target environment [12,13].

Grain yield is the most complex character with quantitative nature, having a diverse environmental effect. Therefore, it is indispensable to take out the selection based on yield stability assessment than average evaluation in different environmental situations [14,15]. Genotype × environment interactions are a considerable barrier for the crops to achieve entire genetic gain [16]. So, the breeding program emphasizes improving the grain yield in a specific environment or a vast range of rice cultivating conditions.

Sharma [17] reported that adaptation is a time-and-place situation to use the capacity of the genotype to support the utilization of the macro-environment. Breeding of location-wise cultivars has been considered the best approach to combat future food crises by enhancing crop production [18]. A true evaluation of the accomplishment of a genotype in different locations is a possible adaptation of its own [19]. The multiple environmental trials aim to detect higher cultivars for diverse situations. Due to unexpected environmental reasons, various models (AMMI, GGE, GEI, GE) are evolved to explain the result of genotype, environment, or interaction, and are now adopted in reproductive research [20,21]. Recently, several works have applied this analysis to detect mega-environments as salinity stress [22].

Thus, standard trial sites, diverse genotypes, and effective statistical tools such as the sum of squared deviations from regression [23], stability variance [24], coefficient of determination [25], coefficient of variability [26], and additive main effects and multiplicative interaction (AMMI) [27,28] are required.

GGE-biplot was recommended by Yan et al. [12] for a graphical depiction of GEI de-sign of multi-environment data, and it has a lot of advantages. G (genotype main effects) and G E (genotype-environment interaction) are used in biplot to examine genotype and environment simultaneously [29]. Through data visualization, a “which-won-where” view of the biplot assists to detect the best cultivars in the relevant environments [12,30]. Many researchers used the AMMI and GGE biplot approaches extensively to determine the effect of genotype, environment, and their interaction (GEI) on grain yield [31,32,33]. Grading of environment and genotypes average that the stability and comparison of genotypes with well-known cultivar and analysis of biplot paves the way for further steadfast and accurate data explanation in the multi-environment trial. However, breeders’ attempts are spotlighted on evolving new elite breeding lines that can substitute the existing one in relation to superior salt-tolerant ability, and good grain acceptability with high yield. Besides, effective breeding methods and rapid generation advancement is needed to alleviate the antagonistic impacts of vulnerable climate change [34]. The prior findings show that a total of eight BRRI-developed rice varieties were widely evaluated for their act of adapting consecutively for four years in the Southern coastal areas of Bangladesh across six different environments [15].

Thus, the present research investigates genotype’s yield potential individually, assesses the potential presence of diverse mega environments, identifies the best performing genotype for each mega environment, and the best site as a representative of salinity stress conditions, including selected stable and best genotypes.

## 2. Results

### 2.1. Salinity Levels in the Trial Locations

**Figure 1:**
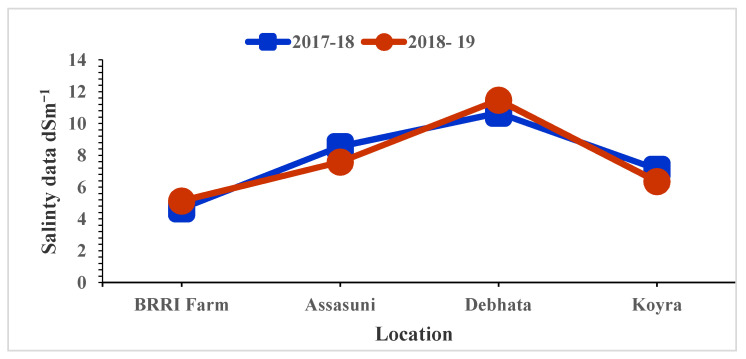
Salinity level of tested entries varied at different locations in coastal areas in Bangladesh.

The high salinity levels were recorded in Debhata compared to Satkhira BRRI Farm. In Debhata, the salinity level of the experimental field varied from 10.67 dS/m to 11.48 dS/m while, 4.65 dS/m to 5.13 dS/m was recorded in the Satkhira BRRI farm (Figure 1).

### 2.2. Trait Characterization of Breeding Lines Using Trait Specific SNPs

All the breeding lines were genotyped and scored 20 trait specific SNP markers linked to the traits for example grain quality [Wx-A_group {snpOS00445 (C)}, Wx-GBSS-ex10 {snpOS00038 (T)} for amylose content; chalk5_576 {snpOS00024 (G)} for chalkiness; Gn1a_1 {snpOS00396 (T)} for grain number; snpOS00397 (T), qSES1-2_2: snpOS00409 (C), qSES1-2_3: snpOS00410 (A), qSES1-2_4: snpOS00411 (T) for salt tolerance; Pi-ta: snpOS00006 (C), Pb1: snpOS00478 (T), Pi33_1: snpOS00468 (T), Pi9_1: snpOS00451 (C) for rice blast; xa5-S1_SKEP: snpOS00054 (AG), xa13_1: snpOS00493 (C), Xa21_SKEP: snpOS00061 (C) for bacterial leaf blight; Bph17_3: snpOS00430 (G), BPH32: snpOS00442 (G), BPH9: snpOS00486 (A) for brown plant hopper; and Gm4_3: snpOS00466 (A), Gm4_4: snpOS00467 (C) for gall midge] from the Intertek (https://www.intertek.com/agriculture/agritech/ (accessed on 26 March 2021) service provider. Based on genotypic data, BR9621-B-1-2-11 harbored 9 QTLs/genes, both BR9620-2-7-1-1 and IR 103854-8-3-AJY1 carried 7 QTLs/genes that control the trait of interest. The rest of the genotypes carry 2–6 QTLs/genes. The 18 breeding lines were characterized using trait-based SNP markers revealing the useful traits with corresponding favorable alleles linked to the specific SNP in Table 1. Since the current investigation is on SNP-based marker-assisted selection thus we have used only 20 trait/gene-based markers. These 20 SNP markers are associated with highly valuable traits (the trait of interest). These trait-linked markers are applied to select the desirable genotypes based on the availability of useful traits or genes.

### 2.3. Stability Analysis by AMMI Model

#### 2.3.1. AMMI Analysis of Variance

From AMMI analysis, the grain yield of 18 breeding lines from six environments explained that the main part of the total sum of the square responsible for environmental impact (62.38%) followed by the GEI effect (19.7%) and the genotypic effect (14.48%) (Table 2). AMMI ANOVA has shown significant differences between 18 genotypes and six environments. It shows that the production of genotype crops of the elite rice is influenced by genotype (G), and environment (E), including interactions between genotype and environment (GEI). AMMI analysis divided GEI into the first two-fold terms of IPCA1 and IPCA2 with a supplement of 52.1% and 19.4% of the total class of GEI. The existence of a substantial percentage of GEI requires a stable analysis of the genotype of rice in the environment.

#### 2.3.2. Grouping of Genotype and Test Locations

**Figure 2:**
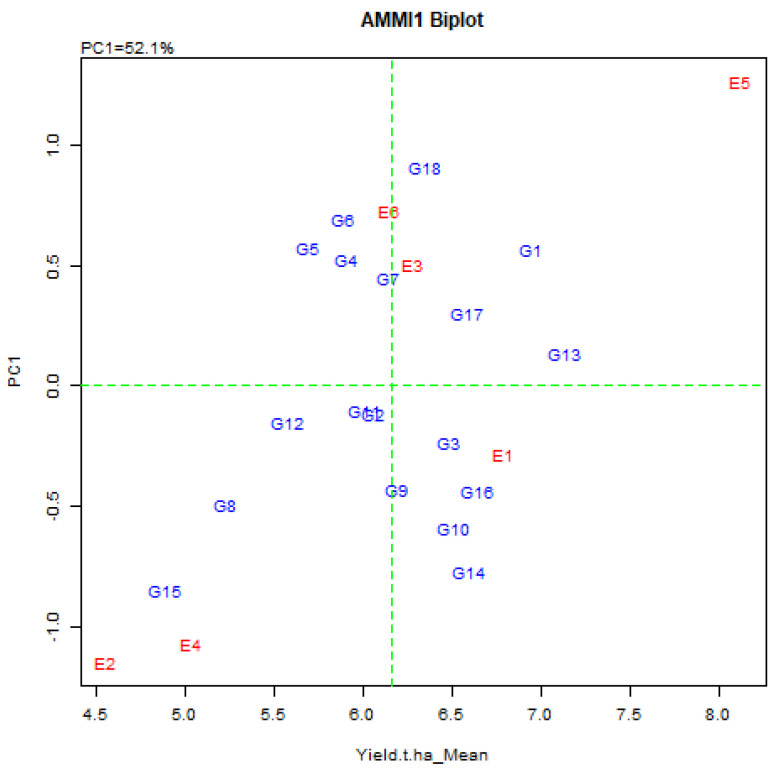
AMMI 1 biplot for grain yield (t/ha) of 18 elite rice genotypes (G) and six environments (E) indicating genotypic and environmental IPCA scores. (First principal component of the interaction = IPCA1, G1 = BR9627-1-3-1-10, G2 = IR92860-33-CMU1-1-CMU2-AJYB, G3 = BR9154-2-7-1-2, G4 = BR9620-2-7-1-1, G5 = BR9620-2-4-1-5, G6 = BR9621-B-1-2-11, G7 = BR9625-4-1-2-8, G8 = BR9625-B-2-4-9, G9 = BR9625-B-1-4-6, G10 = BR9625-3-1-12, G11 = BR9626-1-2-12, G12 = IR 103512-B-AJY 2-2, G13 = IR 104002-CMU 28-CMU 1-CMU 3, G14 = IR 103854-8-3-AJY 1, G15 = IR 103499-B-2-AJY 1, G16 = BRRI dhan28 (Sus. Ck.), G17 = BRRI dhan67 (Res. Ck.) G18 = Binadhan-10 (Res. Ck.)).

**Figure 3:**
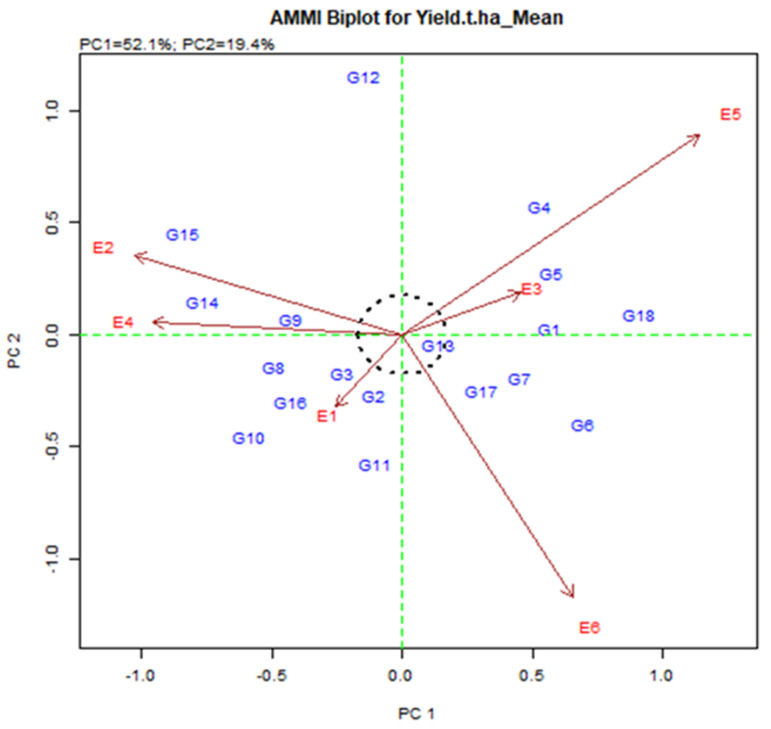
AMMI 2 biplot for grain yield (t/ha) exhibiting the interrelation of IPCA2 against IPCA1 scores of eighteen elite rice genotypes in six environments in Bangladesh.

Biplot analysis is probably the utmost effective explanation tool for the AMMI model and in a biplot graph, both the genotype and the environment are plotted on a similar axis to visualize the interconnection. The AMMI biplots have two elements, the AMMI 1 and AMMI 2. In AMMI 1 biplot, the main impact (genotype means and the environment means) and the IPCA1 score for both genotype and environment are plotted opposite to each other (Figure 2). In AMMI 2 biplot, the score is plotted for IPCA1 and IPCA2 (Figure 3). Grain yield of rice exposed that the environmental means extended from 4.55 ± 0.69 t/ha (Debhata) to 8.13 ± 1.03 t/ha (Satkhira BRRI farm 2017–18), where salinity level was high to low. Eight genotypes exhibited above-average yield in the E1 environment. E2 has exceeded the average yield of eight genotypes, 11 in E3, 8 in E4, 9 in E5, and 10 in E6 environments in a particular environment (Table 3).

The grain yield of eighteen breeding lines varied from 5.18 to 6.94 t/ha. The highest grain yield was G1 and the lowest was G6. The average crop yield on the environment and genotype is 6.16 t/ha. Based on the environmental indicator value from the positive and negative sides, E2, E4, and E6 are bad and E1, E3, and E5 are favorable environments. Genotypes G1, G4, G4, G7, G9, G10, G13, G14, G17, and G18 have high average yields and adapt to this genotype favorable environment, when genotypes G2, G3, G5, G6, G111, G12, G15, and G16 have below-average yield than the mean yield. Moreover, the environment was highly productive when there was high heritability for yield.

#### 2.3.3. AMMI Stability Value (ASV)

The distinctness of the stability assessment of the two main elements can be reimbursed by a proportional contrast between the IPCA (1:2) and then decided by the Pythagoras theorem under the influence of the AMMI stability value. The AMMI Stability value (ASV) is neither measuring quantitative parameters according to their yield stability. Interaction is the main factor in one (IPCA1) score and the AMMI model’s interaction main element is the two (IPCA2) stability indicators. The genotype G15 was the most stable genotype in terms of the first interaction principal component (IPCA1), with an IPCA1 value of (−0.84), followed by G14, G10, G8, and G16 (−0.77, −0.59, −0.49 and −0.43). When the second interaction principal component (IPCA2) was identified, G11 was the most stable genotype with an IPCA2 value of (−0.58), followed by G10 and G6 with IPCA2 values of (−0.45) and (−0.45), respectively (−0.40). Although the two major components are both intense, the AMMI Stability Value (ASV) is a reliable stability parameter [35]. The genotype with a low ASV value is regarded as stable, while one with a high ASV value is considered unstable. Based on ASV, genotype G13 was the most stable, with an ASV value of 0.38, followed by genotypes G2, G11, which had ASV values of 0.40 and 0.64 in yield, respectively, and genotypes G18, G15, and G14, which had ASV values of 2.45, 2.31, and 2.08 in grain yield (Table 3).

#### 2.3.4. Yield Stability Index (YSI)

In this case, ASV considers both IPCA1 and IPCA2 and supports utmost of the diversity of GEI. When the value of YSI is found least then the genotype is deliberated as the most steady and superior with a high mean yield. Based on YSI, the high yield with the utmost stable genotypes are G14 and G18 with the value of YSI 5 chased by G1, G10, G4, and G7 with the value of YSI 9, 11, 12, and 19 respectively (Table 3).

#### 2.3.5. AMMI 1 Biplot Display

In the AMMI biplot, the abscissa consistently represents the difference between the displaced main (additive) effects, whereas the coordinates consistently indicate the difference in the displacement interaction effect As a result, genotypes G1, G13, G17, and G18 usually show high yields with high principal (addition) effects, but genotype G13 is the overall best (Figure 2).

#### 2.3.6. AMMI 2 Biplot Display

In AMMI 2 biplot (Figure 3), the environmental score is added by the sideline. Short-spoking sites do not apply powerful interactive force. Those who have long spokes apply strong interactions. Figure 3 is associated with the origin of the dots representing the environment E1, E2, E3, E4, E5, and E6. The environment was E1 and E3 were short, and they do not apply strong interactive energy nevertheless the environment E5 and E6 were long spoke and therefore symbolize the most discriminatory environment. The genotype on the plot will produce the same in all environments, although the genotype may either be distinct in yield production or exhibit a diverse pattern of environmental reactions. Therefore, the genotype near the origin is not precise to environmental interactions and those who are far from the origin are sensitive and have large interactions.

### 2.4. Stability Analysis of GGE Model

#### 2.4.1. GGE Analysis of Variance

GGE biplot analysis detected 6 principal components (PCs) and all PCs had significant variation (Prob. F < 0.01). PC_1_ and PC_2_ contribute 82.5% and 8.6% to the total variation, respectively where GGE analysis could explain 91.1% variation (Table 4).

#### 2.4.2. Relationship among Testing Environments

**Figure 4:**
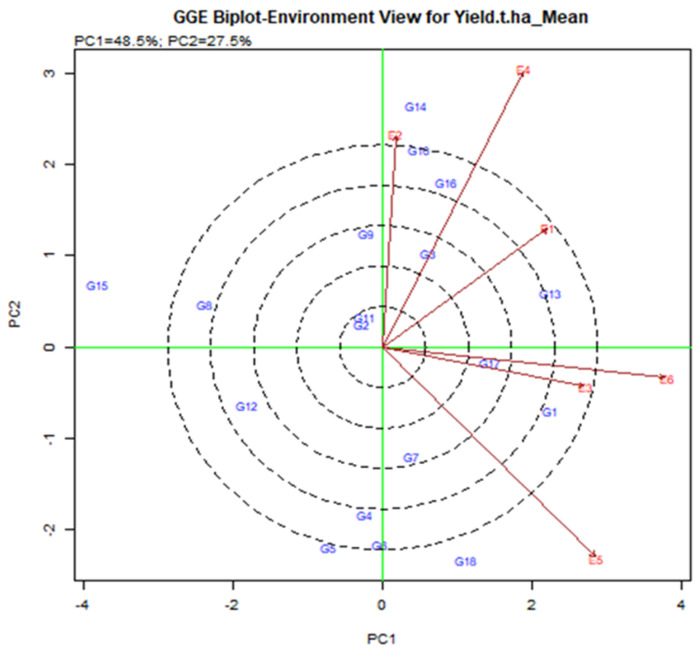
Interrelation among the tested environments of elite rice genotypes for yield was assessed transversely across six environments in Bangladesh.

In the current study, the GGE biplot analysis shows that PC1 and PC2 are responsible for the GGE sum of 48.5% and 27.5% square respectively, explaining 76% of the total variance. The lines that are connected to the origin of the biplot are called vectors. A long vector means that the genotype performance is greater. The E4, E5, and E6 were the longest vectors and making them more discriminatory than other environments (Figure 4). The environment is less useful with very small vectors because there is little discriminating information about the genotype. The test is drawn to connect the environment to the biplot source called the environment vector to visualize the interrelationship between the environments. The relationship partner is proportional to the cosine of the angle between the two surroundings [36]. From Figure 4, E2 and E4 were positively related (an acute angle), E2 and E5 were negatively related (an obtuse angle) and E1 and E5 were not related (a right angle). Strong negative relationships between the tested environment mean that is stronger by G × E interaction. E2 and E5 have larger G by E due to the larger crossover among all environments. According to the angle of the tested environment, six environments are divided into two groups. A group that included E1, E2, and E3 was closely related, i.e., it provides almost the same information.

#### 2.4.3. Identification of Ideal Environments

**Figure 5:**
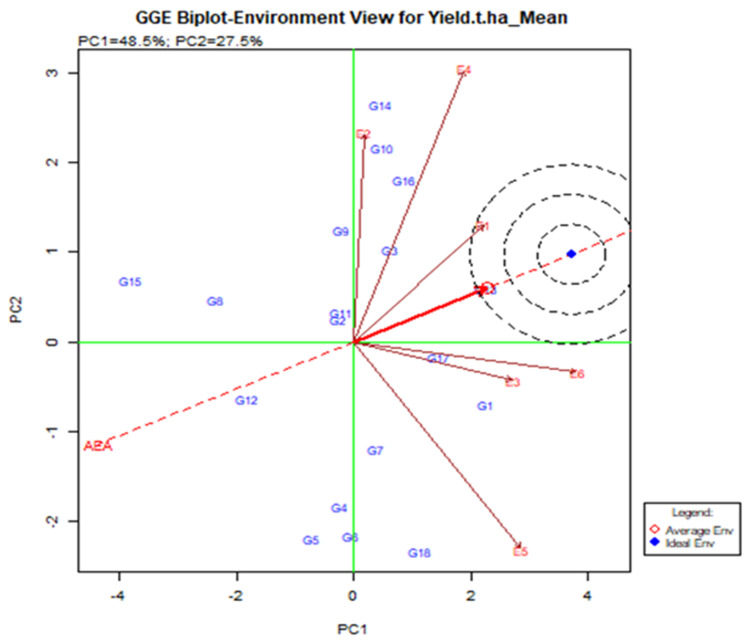
Relationship among the test environments constructed on the average environment axis (AEA), regarding stability and adaptability of elite rice genotypes for grain yield estimated over six environments.

A line that crosses the origin of the biplot is called the Average Environment Axis (AEA). This indicates a nearly favorable situation for the tested genotype. In Figure 5, E1 is most representative of the small angles with AEA where the adapted genotype is usually used. E2 and E5 are the least representative where genotype is used specifically for that environment. The ideal test is the center of the eco-centric circle. E1 indicates the best environment for this point and generally adapted genotype across the environment when E2, E4, and E5 are still there.

#### 2.4.4. Rice Genotypes’ Performance and Stability in a Variety of Tested Environments

**Figure 6:**
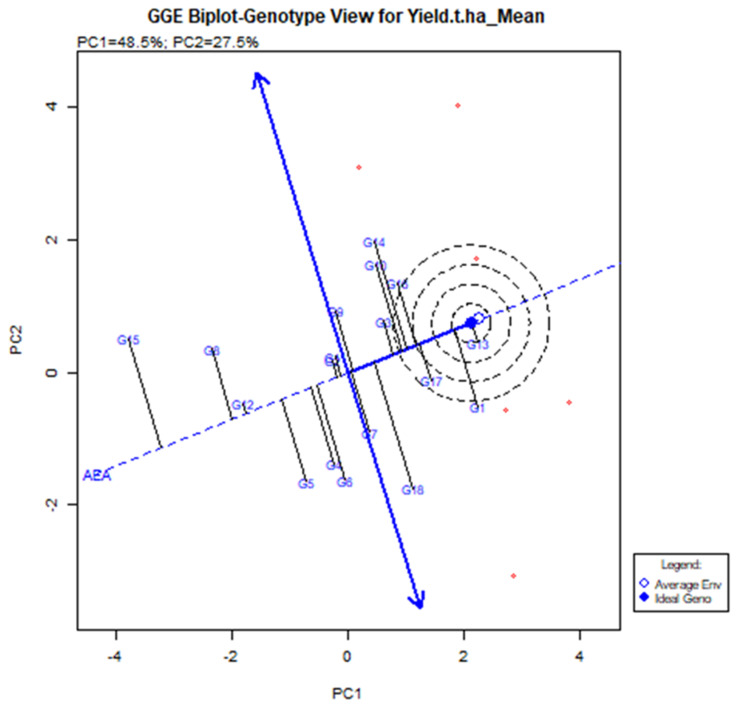
The mean and stability of elite rice genotypes for yield and definite genotype-environment interrelations during two consecutive seasons.

The GGE Biplot visualization is useful in terms of executing and locating the best stable genotype [37]. When compared to genotypes located outside, more stable genotypes placed in the concentric area yielded more, but the environmental impact was too great. G13 was the model genotype based on the GGE biplot (shown by the brave point in the center of the center). The G13 produced the highest yield based on an average of multiple conditions, and it is defined by its position at the far-right end of the AEA line. The average yield of genotype G13 was 6.62 t/ha (Table 3) with vector deviation from the origin point indicating the instability of the genotypes (Figure 6). Five genotypes are set up in the central areas, such as G1, G10, G14, G16 (BRRI dhan28), and G17 (BRRI dhan67). G17 (BRRI dhan67) yields above average and is relatively stable.

However, G13 (IR 104002-CMU 28-CMU 1-CMU 3) had comparatively higher yields than BRRI dhan67 and it was significantly higher than the BRRI dhan28. G1 (BR9627-1-3-1-10) had the highest yield of 6.62 t/ha, although G18 (Binadhan-10), G17 (BRRI dhan67), and G16 (BRRI dhan27) had 6.60, 6.24 and 6.12 t/ha (Table 3). The second maximum yield was G14 (IR 103854-8-3-AZY1) average production was 6.68 t/ha. However, the vector line’s aberration was too high, and the vector line’s end was placed outside the threshold line. This suggests that the G14 was not generally adopted, and the yield was not consistent across the board.

#### 2.4.5. Identification of Which-Won-Where and Mega-Environment (Mega-E)

The exhibition of which-won-where multi-environment yield trial data is critical for determining the presence of many mega environments in the target environment [12,30]. The polygon feature of a biplot is the most popular way to depict genotype-environment interaction patterns [29] to reveal the existence or missing of cross over GEI which is useful in judging the probable presence of various mega environments [10,30,31].

**Figure 7:**
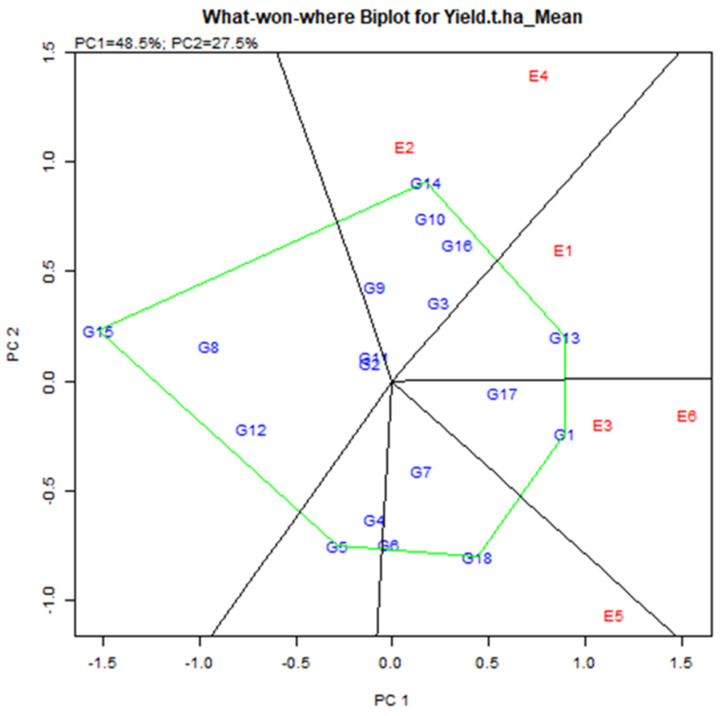
Mega-environments with winning genotypes during two consecutive seasons.

Environmental group Mega-E has an affinity to help the accomplishment of several genotypes concurrently [38]. Mega-E vertex determines by genotype, i.e., GGE analysis visualizes the maximum yield genotype in each quadrant [39]. The link line, which begins at the biplot’s base and crosses individual associating lines to divide the biplot into many sectors, was used to connect the vertex’s locations. Environment integrated sector, i.e., the Environment represents a point-of-view sector, called Mega-E [40]. It consists of six main quadrants based on vertex genotype and vector line cross (Figure 7). Two mega in six quarters. Mega-E1 consisted of E2 (Debhata 2018–19) and E4 (Satkhira Farm 2018–19). Mega-E2 consisted of E3 (Sathkira Farm 2017–18) and E6 (Koyra 2018–19). Hence, the winning genotype in Mega-E was G14 (IR 103854-8-3-AJY 1) for the first; G1 (BR9627-1-3-1-10) for the second environment (Figure 7). Also, the genotype in the polygon (for example G10, G16 for Mega-E1) was less sensitive to the position compared to the winning genotype [41].

### 2.5. Correlation Coefficient of Grain Yield in Different Environments

**Figure 8:**
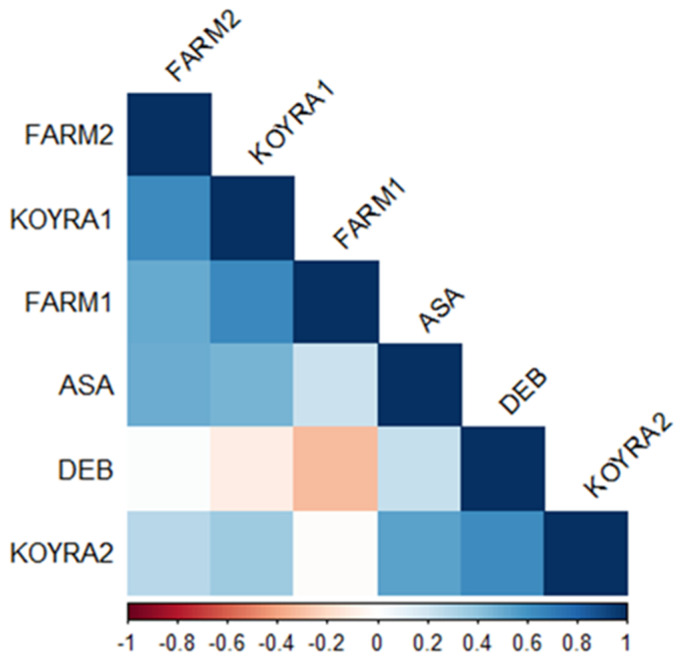
Heatmap of linear Pearson’s correlation coefficients between grain yield tested in diverse environments (ASA = Assasuni 2017–18, DEB = Debhata 2018–19, KOYRA1 = Koyra 2017–18, KOYRA2 = Koyra 2018–19, FARM1 = Satkhira BRRI farm 2017–18, FARM2 = Satkhira BRRI farm 2018–19).

The correlation coefficients for grain yield were no-ticed for the following pair of environments because they were statistically significant (α = 0.05): Assasuni 2017–18 to Koyra 2018–19 (r = 0.54), Assasuni 2017–18 to Satkhira BRRI farm 2018–19 (0.50), Debhata 2018–19 to Koyra 2018-19 (0.62), Koyra 2017–18 to Satkhira BRRI farm 2018–19 (0.63), Koyra2017-18 to Satkhira BRRI farm 2017–18 (0.65), and Satkhira BRRI farm 2017–18 to Satkhira BRRI farm 2018–19 (0.50) (Figure 8). Nevertheless, negative correlation coefficients were detected for grain yield in Debhata 2018–19 to Satkhira BRRI farm 2017–18 (−0.32), Debhata 2018–19 to Koyra 2017–18 (−0.10) as well as Koyra 2018–19 to Satkhira BRRI farm 2017-18 (−0.01) (Figure 8).

### 2.6. Cluster Analysis

**Figure 9:**
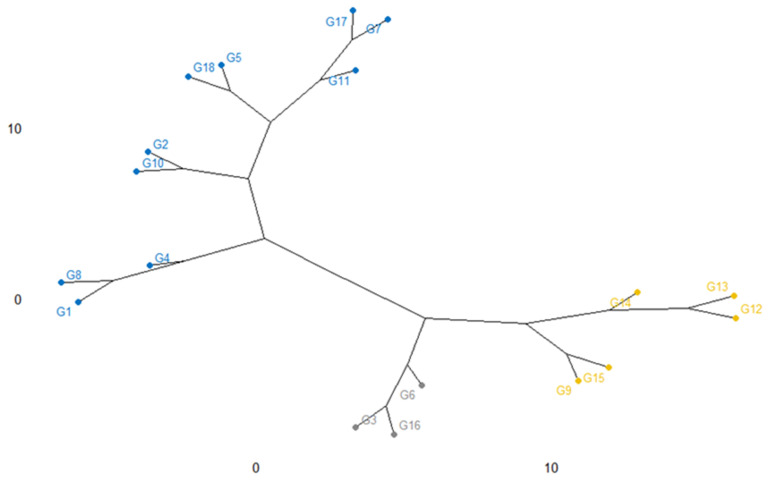
Cluster analysis showing the genotypic diversity and relevancy along with the 18 salinity tolerant rice genotypes based on yield traits.

The relatedness among the eighteen rice genotypes as characterized by dendrogram utilizing the agglomerative cluster method is shown in Figure 9. In this cluster, genotypes were grouped into three clusters. Cluster I consisted of 10 accessions e.g., G4, G1, G8, G2, G10, G5, G18, G11, G7, and G17; Cluster II includes 5 accessions namely, G9, G15, G14, G12, and G13 although cluster III consisted of others 3 genotypes (G6, G3, G16) noticing that the clustering of the elite genotypes is occupying on the parental evolutionary basis.

The dendrogram exhibited that a higher genetic distance exists between Cluster I and Cluster III noticed that having a different background of genetic material. In cluster I, G4 (BR9620-2-7-1-1) and G17 (BRRI dhan 67) had the highest diversity, which was apparently due to the diverse parents by producing productive outputs or may be in the interest of a kind of mutation. Although, G7 (BR9625-4-1-2-8) was identical to the G17 (BRRI dhan 67). Most of the time, the divergence of genotypes among them may depend on the variation of their parental outputs. Otherwise, clusters II, G9 (BR9625-B-1-4-6), and G13 (IR 104002-CMU 28-CMU 1-CMU 3) are dissimilar, even though they are existing the similar species, however the parents responsible for the theory of crosses were distant and may have a dissimilar origin of species.

## 3. Discussion

In Bangladesh, the dry or boro season is critical for increasing rice production and maintaining food security because this is the season when the largest yield is acquired to meet the target production. Soil and water salinity are the most important environmental factors, and there is a strong negative relationship between yield and salinity [15]. High yield was acquired in the non-saline or trace saline levels in BRRI farm (EC: 4.65 to 5.13 dS/m), Debhata (10.67 to 11.48 dS/m), Assasuni (7.58 to 8.56 dS/m) and Koyra (6.36 to 7.13 dS/m). It is suggested to check the suitability and adaptability of new genotypes across hotspot areas with different findings [15].

Since its release in 1994, BRRI dhan28 has been one of the most popular kinds for the boro season in Bangladesh. The adverse effect of salinity on the yield assessment of BRRI dhan28 was found. In saline-prone locations, however, the yield performance of BRRI dhan28 (a salt-susceptible variety) was not the poor yielding genotype. BRRI dhan28 produces a poor yield in these salinity-affected areas, but farmers love it for its great grain characteristics (medium slender grains), flavor (eating and cooking quality), shorter growth length, and good market price. It has been noted that the early maturity of BRRI dhan28 may help this cultivar partially avoid salt stress during the reproductive (booting/flowering) stage. The finding of some elite breeding materials that produced higher yields than BRRI dhan28 and other salt-tolerant cultivars BRRI dhan67 and Binadhan-10 was a valuable indictment from this study.

### 3.1. Discussion Based on AMMI

Significant G × E interaction means that there was a notable difference between the grain yield of crops in six different environments. The majority of total diversity is explained by the major environmental impact that is reflected in the environmental effect and a major part of crop production. The most important genotype for crop production is the environmental interaction effect that ensures that the genotype responds diversely to the diversity of environmental situations. The yield diversity can be featured in the diverse environmental (climatic) situations and diverse edaphic circumstances at diverse locations. So, the analysis of stability and adaptability is essential for the detection of widely or precisely adapted rice genotypes. Similar output was found by [18,42,43].

In addition, a high-yielding genotype with high heritability is useful for selection. This statement confirms in wheat [44]. However, the lower heritability for grain yield in salt stress indicates that yield is very affected by salinity, and environmental factors. In other words, the genetic sensuous signals of low broad-sense heritability for yield in salinity stress conditions indicate that there was not enough genetic difference in the oversight of grain yield in the tested genotype. Stability is neither the only attribute for selection, as the most stable genotype will not essentially provide the outstanding yield execution [45]. So, a single index requires a mechanism to include both yield and stability, so different authors have also recommended diverse selection ethics for the selection of yield and stability: rank-sum, modified rank-sum, and statistical stability [46,47]. Moreover, YSI is implemented to detect high-yielding stable cereal crop genotypes such as maize [48] and durum wheat [45]. Utilizing these steps, the appropriate rice genotype can be recognized for various environmental situations.

The higher value in IPCA 1 was enough to investigate the total G × E interaction that was supported by different investigators in various crops like rice mungbean, maize, bread wheat, finger millet, and common bean [49,50,51,52,53,54].

Genotypes that have similar adaptations to a group when a group affects the genotype in a similar pathway [55]. Best adaptable can plot far away from genotype. If the IPCA1 score in a genotype or environment is almost zero, it has a little interrelation effect and is considered stable. When a genotype and environment have similar marks on the PCA axis, their interrelation is positive and if different, their interrelation is negative. The same parallel line has a genotype and environment, relative ordinate similar yield, and a genotype on the right side of the middle point of the axis, or the environment is more productive than the left.

Therefore, genotype G13 is specifically identified as environment E3 and E5 adaptive. This environment is considered to be a wide range of favorable environments for that genotype. This is steady with earlier reports accredited to [56,57]. Meanwhile, the environment E3 and E5 had positive IPCAl scores and are far from zero and so all the genotypes performed well in this position that indicating the medium to large interaction effect. The results agreed with the interpretation of rice and sorghum [58,59].

Similarly, favorable environments for (E1) genotype G3, G9, G10, G14, and G16 have been found with negative IPCA1 scores but higher yields than average output. Besides, genotypes G2, G8, G11, G12, and G15 were stable across the environment below average production (low negative IPCA1 score). Genotype alongside negative IPCA1 score near zero indicated that this species was less affected by the environment. Besides, the genotype was moderately stable across the G4, G5, G6, and G7 environments (low positive IPCA1 scores) and under average production.

In short, The AMMI 1 biplot is utilized to determine the G × E interaction pattern of rice production. Genotypes G1, G13, G17, and G18 are rarely afflicted by G × E interactions and will execute well transversely in different environments. Locations, by means of E1 and E3, may be considered the best selection location for rice development because of stable yields. Stability indicates the key to selection with high grain yield [60]. ASV plays an important role in selecting a stable genotype which may not highest yield always but the selection of genotype based on ASV performs consistent adaptability and yield stability. Odewale et al. [61] described the five coconut genotypes evaluated across nine locations in southern Nigeria. Two coconut genotypes out of five genotypes exposed lower ASV and best stability. Furthermore, Farshadfar [46] assessed the 20 bread wheat genotypes, and three genotypes out of 20 exhibited low ASV and high grain yield compared with the grand mean and therefore relatively better stability. But Lule et al. [53] informed three genotypes out of 32 of finger millet that had higher grain yield, although, with higher ASV and so better genotypes with higher yield accomplishment need to more evaluated for yield and precise adaptability. In the current study, genotypes G4, G11, and G12 were more reactive since they were far from the origin while genotypes G1, G13, G14, and G18 were near to the origin and as a result less responsive to environmental interaction. G1 was very nearby to the origin, so it is stable on the way to the environment. For multifaceted perspectives, The AMMI model is good for the division between G × E diversity, which is used to identify environmental latent and to recognize higher genotypes with precise adaptations or enhanced adaptations [55,58].

### 3.2. Discussion Based on GGE

The discriminatory potential of GGE and representational opinion—A comprehensive assessment of the biplot test setting [11]. The circumference of the circle centered on the biplot predicts the circumference of the environment vector, which depicts the proportional and environmental lysing of the ideal deviation stoicism with the biplot’s environment [31].

Current research has shown that the environment E4 and E5 is obtained with the largest vector than others having discriminating force. The average environment axis (AEA) view compares to an ideal environment. The environment E1 had the narrowest angle with the AEA, therefore environment E1 is highly concentrated. In terms of discrimination and representation, the typically adapted genotype-environment was selected from E1, while the specially adapted genotype-environment was selected from E4 and E5. These types of discrimination and representation findings were found in various studies [62,63,64].

Kaya et al. [44] explained the existence of adjacent relationships within the test environment and concedes that very much alike information around genotypes is available in less experimental environments and therefore there is a good chance of reducing test costs under limited resources. Otherwise, E4, E5, and E6 provide negative relationships, i.e., larger G by E which helps for reproductive selection and efficiency.

Yan and Tinker [31] explain that the narrow-angle of the two arrows means a close relationship between the two environments. Because of the stronger impact of GE on the experimental properties, the larger angle of the two arrows indicates that the results are more divergent.

In the GGE biplot, G3 (BR9154-2-7-1-2) and G17 (BRRI dhan67) are the most stable genotypes, which are closely indicated in the AEA line with too few deviation vectors. It indicates that these genotypes deceived the widespread adaptable and stable one crosswise the environment contrasted to other 16 genotypes. Akmal et al. [65] exhibited that the average higher yield genotype was not the most stable, and vice versa.

Highly stable genotypes such as G13, G13, G11, G14, G14, G16, and G17 showed less unmarked and good production capacity. The genotypes G5, G8, and G15 were extremely unstable as it is located far away from the centric AEA line, which is shown below the average yield capacity. The findings have based the explanation of various crops like mungbean [62,64]. Based on the which-won-where biplot analysis, G13 for E1, G14 for E2 & E4, G1 for E3 & E6 and G18 for E5 were the vertex cultivars to reveal the best yield capabilities. These types of potential yield effectiveness of vertex genotypes in the tested environment are described in mungbean [62,64], which is agreed upon in the present study.

### 3.3. Cluster Analysis

Genotype diversity is an elementary for genetic makeup advancement, especially for rice plants. The expertise of genotype or genetic relatedness among genotypes supplies helpful information to forward the breeding and germplasm enhancement programs [66]. Characterization of collected germplasm is the conventionally essential basis on phenotypical and agronomical characters, which is highly appreciable for plant breeders or workers. Cluster-I contains 10 genotypes and cluster-II and cluster-III have five and three genotypes respectively. This signifies that crossing between elite genotypes of the mentioned diverse clusters might supply enticing recombinants for evolving high-yielding rice varieties. This type of complementary work was accounted for by Verma et al. [67], who categorized 108 wheat genotypes into eleven clusters, Khodadadi et al. [68] divided thirty-eight wheat breeding lines into seven clusters, Tsegaya et al. [69] divided twenty-one local wheat genotypes into six clusters, Alemu et al. [70] divided sixty-four breeding lines of durum wheat consists of twelve clusters. Therefore, genotypes from diverse clusters should be selected for hybridization in rice breeding programs. Commonly, these rice genotypes in this finding showed higher diverse genetic variability between them. This type of finding was found by Singh and Upadhyay [71] highest cluster interval shows a wide extent of genotype diversity and finally may be utilized belongs to the variety development program.

## 4. Materials and Methods

### 4.1. Genotyping for Trait Characterization

Eighteen breeding lines were genotyped with 20 trait-based single nucleotide polymorphism (SNP) markers evolved by the International Rice Research Institute (IRRI; https://www.irri.org/ (accessed on 7 April 2021); https://gsl.irri.org/ (accessed on 10 April 2021); [72]) utilizing Kompetitive allele-specific PCR (KASP) assay for high-precision bi-allelic characterization of SNP with Intertek (https://www.intertek.com/agriculture/agritech/ (accessed on 26 April 2021) as a service provider. The SNP markers associated with the trait of interests such as snpOS00445, snpOS00038 for amylose content; snpOS00024 for chalkiness (grain quality); snpOS00396 for grain number; snpOS00397, snpOS00409, snpOS00410, and snpOS00411 for abiotic stress salt tolerance snpOS00006, snpOS00478, snpOS00468, snpOS00451 for rice blast; snpOS00054 (AG), snpOS00493, snpOS00061 (C) for bacterial leaf blight; snpOS00430, snpOS00442, and snpOS00486 for brown planthopper; and snpOS00466, snpOS00467 for gall midge were assayed.

These 20 SNP markers are linked with highly important traits (the trait of interest). These trait-linked SNP markers are used to identify the appropriate genotypes based on the availability of desired traits.

### 4.2. Genotypes Testing Locations

**Figure 10:**
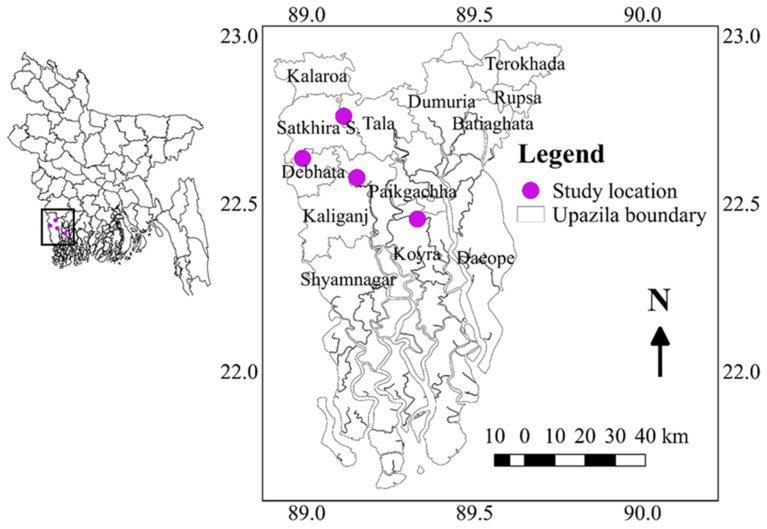
Geographical location of the experimental sites.

The salinity level was measured in the main field at seven-day intervals from the time of transplanting to the flowering time of the all-tested genotypes using a portable electrical conductivity (EC) meter (HANNA, HI8733). To evaluate the genotypes, the experiment was conducted in six diverse environments in the southern part of Bangladesh such as Assasuni 2017/18 (E1) Debhata 2018/19 (E2), Sathkira BRRI Farm 2017/18 (E3), and 2018-19 (E4) at Satkhira district; and Koyra 2017–18 (E5), Koyra 2018–19 (E6) at Khulna districts representing different salinity level tolerances. Sathkira BRRI farm, Koyra, and Assasuni, Debhata are favorable, medium-stress, and high-stress sites, respectively (Figure 10). The monthly mean temperatures, wind speed, and precipitation data were presented in Table 5.

### 4.3. Plant Genetic Materials

Eighteen genotypes consisting of fifteen advanced breeding lines (BRRI developed ten lines: BR9627-1-3-1-10 (G1), BR9154-2-7-1-2 (G3), BR9620-2-7-1-1 (G4), BR9620-2-4-1-5 (G5), BR9621-B-1-2-11 (G6), BR9625-4-1-2-8 (G7), BR9625-B-2-4-9 (G8), BR9625-B-1-4-6 (G9), BR9625-3-1-12 (G10); and five lines from International Rice Research Institute (IRRI): IR92860-33-CMU1-1-CMU2-AJYB (G11), IR 103512-B-AJY 2-2 (G12), IR 104002-CMU 28-CMU 1-CMU 3 (G13), IR 103854-8-3-AJY 1 (G14), IR 103499-B-2-AJY 1 (G15), and three checks varieties such as BRRI dhan28 (G16), BRRI dhan67 (G17), and One BINA released variety; Binadhan-10 (G18) were used.

### 4.4. Design of Experiment and Agronomic Practices

The field experiment was evaluated in the dry (*boro*) season in 2017-18 (Y1) and 2018-19 (Y2). At different locations, the sowing of seeds was done from mid-November to the last week of November, and transplantation was completed within the third to fourth week of December in both seasons. Seedlings were uprooted when reached forty days and transplanted maintaining a spacing of 20 × 15 cm with 2–3 seedlings per hill. The unit size of the treatment plot was 5.4 m in length × 10 rows (10.8 m^2^). The layout followed a randomized complete block design with three replications. The crop was fertilized by applying Urea-TSP-MoP-Gypsum-Zinc sulfate at the rate of 120, 19, 60, 20, 3.6 kg of N, P, K, S, Zn, ha^−1^, respectively. At the time of final land preparation, all fertilizers except urea were applied as basal. Urea was applied in three equal splits (at 10–15 days after transplanting, maximum tillering stage, and around one-week s before panicle initiation). The agronomic practice of crops such as weeding, irrigation, etc. was executed on time. Insect pests, diseases, and other pests like rodents, and birds were controlled appropriately. At the physiological maturity stage, the crop was harvested from an area of 10.2 m^2^. To avoid the border effect two border rows were not considered for the yield data measured. The final grain yield (t/ha) data was adjusted at a 14% moisture level and other data were collected pursued by standard calculation method as demonstrated by Yoshida et al. [73].

### 4.5. Statistical Analysis

For grain yield, the combined analysis of variance (ANOVA) was used to determine the effects of environment (E), genotype (G), and their interactions for eighteen breeding lines in six environments. Statistical software STAR (version 2.0.1) was used for the combined analysis of variance and standard error of the mean. Principal component analysis was used to construct the GGE biplot, and PB Tools software (version 1.3; http://bbi.irri.org/products (accessed on 10 April 2021)) was used to create broad-sense heritability for individual trials. The genotypic index (GI) is calculated as (the mean of all varieties at the jth environment—The grand mean) and the environmental index (EI) is calculated as (the mean of all varieties at the jth environment—The grand mean) (the mean of all environments of the ith genotype—The grand mean). Pearson’s correlation coefficients were used to assess the interrelationships between grain yield observed in various conditions. The R statistical software package was used to conduct all the analyses (version 4.0.2).

### 4.6. AMMI Stability Value (ASV)

ASV was calculated using the AMMI model and estimated for each genotype and individual environment based on the contribution of IPCA1. The AMMI stability value (ASV) alleged by Purchase et al. [74] was calculated as:

ASV = [{(SSIPCA1 ÷ SSIPCA2) (IPCA1score)}2 + (IPCA2score)2]1/2

Here, the IPCA1 and IPCA2 scores were the genotypic scores in the AMMI model; SS stands for the sum of squares, while IPCA1 and IPCA2 are the first and second interaction main component axes, respectively. In a two-dimensional scatterplot comparing IPCA1 scores against IPCA2 scores, ASV is the interval from zero. As a result, because the IP-CA1 score contributes more to the GE sum of squares, it must be weighted by the proportional difference between IPCA1 and IPCA2 scores to compensate for the comparative assistance of IPCA1 and IPCA2 total GE sum of squares. The larger the IPCA score (absolute value), additionally negative or positive, a genotype was more distinctly adapted in some environments. Lower ASV quality implies a more stable genotype throughout the environment [74].

### 4.7. Yield Stability Index (YSI)

Yield stability index (YSI) for individual genotype which combines both mean grains yield and ASV index was estimated as YSIi = RASVi + RYi.

Here, the rank of the AMMI stability value for the ith genotype is RASVi, while the rank of the mean grain yield for the ith genotype across environments is RYi [75].

## 5. Conclusions

SNP-based characterization revealed that BR9621-B-1-2-11 (G6) carried the highest number of effective QTLs/genes (9) responsible for high amylose content, higher grain number, and overall phenotypic performance/salt injury score, blast, and gall midge resistance. On the other hand, both BR9620-2-7-1-1 (G4) and IR 103854-8-3-AJY1 (G14) have 7 QTLs/genes that control the desirable traits. However, these 20 important SNPs are helpful to identify the best genotypes for releasing new rice varieties for the farmers of the salt-prone coastal zone of Bangladesh. Moreover, the AMMI analysis demonstrated that among the genotypes G1 (BR9627-1-3-1-10), G13 (IR 104002-CMU 28-CMU 1-CMU 3), G17 (BRRI dhan67), and G18 (Binadhan-10) were shown most promising due to their higher and relatively stable yield. However, GGE biplot analysis illustrated that the genotype G14 (IR103854-8-3-AJY1) was the winning genotype in mega-environment (ME)-I, whereas G1 (BR9627-1-3-1-10) in ME-II. The GGE and AMMI biplot model explains that G1 and G13 are the best genotypes because of the salinity and stability factors, as well as the highest grain yield. Thus these (G1, G6, G13, G14, G17, and G18) improved multiple-stress-tolerant rice genotypes with stable grain yield could be used as elite donor parents for the future breeding program. The genotypes G4 (BR9620-2-7-1-1) and G6 (BR9621-B-1-2-11) and G13 (IR 104002-CMU 28-CMU 1-CMU 3) have a higher number of favorable alleles and stable yield might be released as a new salt-tolerant variety where salinity is the major impediment.

## Figures and Tables

**Table 1:** List of the gene-based SNP markers with favorable alleles used to characterize 18 advanced breeding lines for the different traits of interest.

**Trait**	**Gene/QTL**	**Trait Specific Favorable Allele with SNP Id**	**Genotypes**									
			**BR9154-2-7-1-2**	**BR9620-2-4-1-5**	**BR9620-2-7-1-1**	**BR9621-B-1-2-11**	**BR9625-B-2-4-9**	**BR9625-3-1-12**	**BR9625-B-1-4-6**	**BR9625-4-1-2-8**	**BR9626-1-2-12**	
**Grain Quality**												
Amylose content	*Wx-A_group*	C: snpOS00445	C:C	C:C	C:C	C:C	C:C	C:C	C:C	C:C	-	
	*Wx-GBSS-ex10*	T: snpOS00038	T:T	T:T	T:T	T:T	C:C	T:T	C:C	C:C	-	
Chakiness	*chalk5_576*	G: snpOS00024	A:A	A:A	A:A	A:A	G:G	G:G	A:A	A:A	-	
Grain number	*Gn1a_1*	T: snpOS00396	A:A	A:A	A:A	T:T	A:A	T:T	A:A	T:T	-	
**Abiotic Stress**												
Salt-tolerance	*Saltol-Aus*	T: snpOS00397	G:G	G:G	G:G	G:G	T:T	G:G	G:G	G:G	-	
	*qSES1-2_2*	T: snpOS00409	C:C	C:C	C:C	C:C	T:T	T:T	T:T	T:T	-	
	*qSES1-2_3*	A: snpOS00410	G:G	G:G	G:G	A:A	G:G	G:G	G:G	G:G	-	
	*qSES1-2_4*	T: snpOS00411	T:T	T:T	T:T	T:T	A:A	A:A	A:A	A:A	A:A	
**Biotic Stress**												
Blast	*Pi-ta*	C: snpOS00006	C:C	C:C	C:C	C:C	A:A	C:C	C:C	C:C		
	*Pb1*	T: snpOS00478	C:C	C:C	C:C	C:C	-	C:T	-	C:T	-	
	*Pi33_1*	T: snpOS00468	G:G	G:G	G:G	G:G	G:G	G:G	G:G	G:G	-	
	*Pi9_1*	C: snpOS00451	G:G	G:G	G:G	G:G	G:G	G:G	G:G	G:G	-	
	*xa5-S1_SKEP*	AG: snpOS00054	TC:TC	TC:TC	TC:TC	TC:TC	TC:TC	TC:TC	TC:TC	TC:TC	-	
BLB	*xa13_1*	C: snpOS00493	G:G	G:G	G:G	G:G	G:G	G:G	G:G	G:G	-	
	*Xa21_SKEP*	C: snpOS00061	G:G	G:G	G:G	G:G	C:C	G:G	G:G	G:G	-	
	*Bph17_3*	G: snpOS00430	A:A	A:A	A:A	A:A	A:A	A:A	A:A	A:A	-	
BPH	*BPH32*	G: snpOS00442	-	C:C	C:C	C:C	G:G	C:C	C:C	C:C	-	
	*BPH9*	A: snpOS00486	C:C	C:C	A:A	A:A	C:C	C:C	A:A	C:C	-	
Galmidge	*Gm4_3*	A: snpOS00466	G:G	G:G	G:G	G:G	G:G	G:G	G:G	G:G	-	
	*Gm4_4*	C: snpOS00467	C:C	C:C	C:C	C:C	C:C	G:G	C:C	C:C	G:G	
Number of desirable traits present in each genotype			6	6	7	9	6	6	4	5	-	
**Trait**	**Gene/QTL**	**Trait Specific Favorable Allele with SNP Id**	**Genotypes**									
			**BR9154-2-7-1-2**	**BR9627-1-3-1-10**	**IR92860-33-CMU1-1-CMU2-AJYB**	**IR 103499-B-2-AJY 1**	**IR 103512-B-AJY 2-2**	**IR 103854-8-3-AJY 1**	**IR 104002-CMU 28-CMU 1-CMU 3**	**BRRI dhan28**	**BRRI dhan67**	**Binadhan-10**
**Grain Quality**												
Amylose content	*Wx-A_group*	C: snpOS00445	C:C	C:C	C:C	-	C:C	C:C	C:C	C:C	C:C	C:C
	*Wx-GBSS-ex10*	T: snpOS00038	T:T	C:C	C:C	-	C:C	C:C	T:C	C:C	-	T:T
Chakiness	*chalk5_576*	G:snpOS00024	A:A	A:A	A:A	G:A	G:G	G:A	G:G	A:A	-	A:A
Grain number	*Gn1a_1*	T: snpOS00396	A:A	A:A	A:A	A:A	T:T	T:T	T:T	A:A	-	A:A
**Abiotic Stress**												
Salt-tolerance	*Saltol-Aus*	T: snpOS00397	G:G	G:G	T:T	T:T	G:G	G:G	T:G	G:G	-	G:G
	*qSES1-2_2*	T: snpOS00409	C:C	T:T	T:T	T:T	T:T	T:T	T:T	T:T	-	C:C
	*qSES1-2_3*	A: snpOS00410	G:G	G:G	G:G	G:G	G:G	G:G	G:G	G:G	-	G:G
	*qSES1-2_4*	T: snpOS00411	T:T	A:A	A:A	A:A	A:A	A:A	A:A	A:A	T:T	T:T
**Biotic Stress**												
Blast	*Pi-ta*	C: snpOS00006	C:C	C:C	C:C	-	A:A	C:A	A:A	A:A	-	A:A
	*Pb1*	T: snpOS00478	C:C	C:C	C:C	-	?	C:T	?	C:C	-	C:C
	*Pi33_1*	T: snpOS00468	G:G	G:G	G:G	-	G:G	G:G	G:G	G:G	-	G:G
	*Pi9_1*	C: snpOS00451	G:G	G:G	G:G	G:G	G:G	G:G	G:G	G:G	-	G:G
	*xa5-S1_SKEP*	AG: snpOS00054	TC:TC	TC:TC	TC:TC	-	TC:TC	TC:TC	TC:TC	TC:TC	-	TC:TC
BLB	*xa13_1*	C: snpOS00493	G:G	G:G	G:G	-	G:G	G:G	G:G	G:G	-	G:G
	*Xa21_SKEP*	C: snpOS00061	G:G	G:G	G:G	G:G	G:G	G:G	G:G	G:G	-	G:G
	*Bph17_3*	G: snpOS00430	A:A	A:A	A:A	A:A	A:A	A:A	A:A	A:A	-	A:A
BPH	*BPH32*	G: snpOS00442	-	C:C	C:C	-	C:C	G:G	C:C	G:G	-	C:C
	*BPH9*	A: snpOS00486	C:C	A:A	C:C	C:C	C:C	C:C	C:C	C:C	A:A	A:A
Galmidge	*Gm4_3*	A: snpOS00466	G:G	G:G	A:A	-	G:G	G:G	G:G	G:G	-	G:G
	*Gm4_4*	C: snpOS00467	C:C	C:C	C:C	G:G	G:G	G:C	G:G	G:G	-	C:C
Number of desirable traits present in each genotype			6	4	5	2	3	7	5	2	3	6

**Table 2:** Additive main effects and multiplicative interaction (AMMI) analysis of variance for grain yield (t/ha) of 18 elite rice genotypes throughout six different environments in two years.

Source of Variation	df	SS	MS	F Value	*p*-Value	Variability Explained	
						% GE	% TSS
Environment	5	292.68	58.84	151.98	<0.0001	-	62.38
Rep within Env.	6	2.32	0.38	2.85	0.0131	-	0.49
Genotype	17	67.98	3.99	29.63	<0.0001	-	14.48
Env.: Genotype	85	92.42	1.08	8.06	<0.0001	-	19.70
IPCA1	21	48.13	2.29	17.24	<0.0001	52.1	-
IPCA2	19	17.88	0.94	7.08	<0.0001	19.4	-
IPCA3	17	11.26	0.66	4.99	<0.0001	12.2	-
IPCA4	15	9.43	0.63	4.73	<0.0001	10.2	-
IPCA5	13	5.70	0.44	3.30	<0.0001	6.2	-
IPCA6	11	0.00	0.00	0.00	<0.0001	0.0	-
Pooled Error	102	13.76	0.15	-	-	-	2.94
Total	215	469.16	-	-	-	-	-

*p*-value associated with an effect’s F statistic or significant at the 0.01 probability level, % TSS = Percentage total sum of the square; % GE = Percentage (G × E).

**Table 3:** Mean grain yield, AMMI Stability Value (ASV), yield stability index (YSI), Rank based on Best Linear Unbiased Estimation (BLUE), and Rank of ASV of 18 rice genotypes in six testing environments.

**Geno Code**	**Designation**	**Parentage**	**Rank Based on BLUE**	**E1**	**E2**	**E3**		**E4**		**E5**	
G1	BR9627-1-3-1-10	Bhojon/BRRI dhan47//Meikhao	1	7.76 ab	4.34 d–f	7.46 a		5.38 a–e		9.62 a	
G2	IR92860-33-CMU1-1-CMU2-AJYB	IR45427-2B-2-2B-1-1/3 × IR61920-3B-22-2-1(NSIC RC 106)	11	7.25 a–c	4.24 d–f	6.3 a		4.71 b–e		7.09 de	
G3	BR9154-2-7-1-2	Bhojon/Nonabokra	12	6.04 e–g	4.59 cd	6.14 a		4.49 b–e		8.35 a–d	
G4	BR9620-2-7-1-1	AS996/FL478	5	7.62 ab	5.64 a	6.95 a		5.74 a–c		9.4 a–c	
G5	BR9620-2-4-1-5	AS996/FL478	15	7.54 ab	5.32 ab	6.34 a		6.88 a		7.8 b–e	
G6	BR9621-B-1-2-11	BRRI dhan29/FL478//BRRI dhan 47	18	5.52 g	5.29 ab	4.06 b		4 c–e		6.47e	
G7	BR9625-4-1-2-8	Bhojon/Nonabokra//FL378	8	6.93 b–d	5.56ab	6.35a		6.19ab		7.87a–e	
G8	BR9625-B-2-4-9	Bhojon/Nonabokra//FL378	16	7.66 ab	3.81 f–h	6.73 a		5.68 a–d		8.79 a–d	
G9	BR9625-B-1-4-6	Bhojon/Nonabokra//FL378	7	5.88 e–g	3.97 e–h	7.15 a		4.44 b–e		9.49 ab	
G10	BR9625-3-1-12	Bhojon/Nonabokra//FL378	6	7.68 ab	4.5 c–e	6.41 a		4.41 b-e		7.39 d–e	
G11	BR9626-1-2-12	Bhojon/FL478//BRRIdhan 47	13	6.6 c–e	5.2 ab	7.29 a		5.62 a–d		7.62 c–e	
G12	IR 103512-B-AJY 2-2	IR11T252/IR09N522	14	6.53 c–e	3.46 h	6.25 a		4.6 b–e		9.22 a–c	
G13	IR 104002-CMU 28-CMU 1-CMU 3	IR10F388/IR03W134//IR 72046-B-R-3	3	6.38 d–f	3.62 gh	6.33 a		3.66 de		8.54 a–d	
G14	IR 103854-8-3-AJY 1	IR10C135/IR04A115//GSR IR 1-1-Y4-Y1	2	6.59 c–e	3.96 e–h	5.49 ab		3.48 e		8.66 a–d	
G15	IR 103499-B-2-AJY 1	IR11T156/IR05N412	17	6.1 d–g	4.16 d–g	6.51 a		4.64 b–e		8.49 a–d	
G16	BRRI dhan28	BR 6/PURBACHI	10	5.64 fg	4.12 d–g	5.48 ab		4.76 b–e		6.26 e	
G17	BRRI dhan67	IR 61247-3B-8-2-1/BR 36	9	6.44 c–f	4.96 bc	5.87 ab		6.02 a–c		7.85 a–e	
G18	Binadhan-10	IR 42598-B-B-B-B-12/NONA BOKRA	4	8.05 a	5.2 ab	6 ab		5.94 a–c		7.39 de	
En. Mean ± SE				6.79 ± 0.81	4.55 ± 0.69	6.28 ± 0.86		5.04 ± 0.99		8.13 ± 1.03	
En. Index				0.63	−1.61	0.12		−1.12		1.97	
IPCA1				−0.28	−1.14	0.5		−1.07		1.27	
IPCA2				−0.36	0.39	0.21		0.06		0.99	
LSD (0.05)				0.41	0.29	0.98		0.98		0.85	
Heritability				0.97	0.98	0.79		0.86		0.90	
**Geno Code**	**Designation**	**Parentage**	**Rank Based on BLUE**	**E6**	**G M ± SE**	**Genotypic Index**	**ASV**	**Rank of ASV**	**YSI**	**IPCA1**	**IPCA2**
G1	BR9627-1-3-1-10	Bhojon/BRRI dhan47/Meikhao	1	7.1 ab	6.94 ± 0.51	0.78	1.51	8	9	0.56	0.02
G2	IR92860-33-CMU1-1-CMU2-AJYB	IR45427-2B-2-2B-1-1/3 × IR61920-3B-22-2-1(NSIC RC 106)	11	6.45 b–d	6.01 ± 0.36	−0.09	0.40	17	28	−0.11	−0.27
G3	BR9154-2-7-1-2	Bhojon/Nonabokra	12	3.82 g	5.57 ± 0.45	−0.23	0.64	15	27	−0.23	−0.17
G4	BR9620-2-7-1-1	AS996/FL478	5	7.46 a	7.14 ± 0.39	0.27	1.54	7	12	0.53	0.57
G5	BR9620-2-4-1-5	AS996/FL478	15	5.68 d–f	6.59 ± 0.28	−0.26	1.56	6	21	0.57	0.28
G6	BR9621-B-1-2-11	BRRI dhan29/FL478//BRRI dhan 47	18	4 g	4.89 ± 0.30	−0.98	1.90	4	22	0.69	−0.40
G7	BR9625-4-1-2-8	Bhojon/Nonabokra//FL378	8	6.97 ab	6.65 ± 0.23	0.12	1.23	11	19	0.45	−0.19
G8	BR9625-B-2-4-9	Bhojon/Nonabokra//FL378	16	6.87 ab	6.59 ± 0.47	−0.30	1.33	9	25	−0.49	−0.14
G9	BR9625-B-1-4-6	Bhojon/Nonabokra//FL378	7	7.17 ab	6.35 ± 0.57	0.13	1.16	13	20	−0.43	0.07
G10	BR9625-3-1-12	Bhojon/Nonabokra//FL378	6	5.94 c–e	6.06 ± 0.38	0.20	1.65	5	11	−0.59	−0.45
G11	BR9626-1-2-12	Bhojon/FL478//BRRI dhan 47	13	6.59 bc	6.49 ± 0.26	−0.24	0.64	16	29	−0.1	−0.58
G12	IR 103512-B-AJY 2-2	IR11T252/IR09N522	14	5.38 ef	5.91 ± 0.54	−0.25	1.23	10	24	−0.15	1.16
G13	IR 104002-CMU 28-CMU 1-CMU 3	IR10F388/IR03W134//IR 72046-B-R-3	3	5.6 ef	5.69 ± 0.51	0.46	0.38	18	21	0.14	−0.04
G14	IR 103854-8-3-AJY 1	IR10C135/IR04A115//GSR IR 1-1-Y4-Y1	2	7.15 ab	5.89 ± 0.51	0.52	2.08	3	5	−0.77	0.15
G15	IR 103499-B-2-AJY 1	IR11T156/IR05N412	17	6.94 ab	6.14 ± 0.44	−0.68	2.31	2	19	−0.84	0.46
G16	BRRI dhan28	BR 6/PURBACHI	10	5.09 f	5.23 ± 0.20	−0.04	1.20	12	22	−0.43	−0.30
G17	BRRI dhan67	IR 61247-3B-8-2-1/ BR 36	9	5.98 c–e	6.19 ± 0.27	0.08	0.85	14	23	0.30	−0.25
G18	Binadhan-10	IR 42598-B-B-B-B-12/NONA BOKRA	4	6.47 b–d	6.51 ± 0.30	0.44	2.45	1	5	0.91	0.09
En. Mean ± SE				6.15 ± 1.06	6.16						
En. Index				−0.01							
IPCA1				0.72							
IPCA2				−1.3							
LSD (*p* < 0.05)				0.38							
Heritability				0.98							

Here, E1 = Assasuni 2017–18, E2 = Debhata 2018–19, E3 = Koyra 2017–18, E4 = Koyra 2018–19, E5 = Sathkira Farm 2017–18, E6 = Satkhira Farm 2018–19. Different letters (a–h) within a column denote significance based on LSD test (*p* < 0.05).

**Table 4:** Analysis of variance of principal components of biplot genotype and environment of the *Boro* elite rice genotypes in six environments.

Principal Component	Effect (%)	Accumulated Variance (%)	DF	SS	MS	F Value	Pr. F
PC1	82.5	82.5	21	4773.38	227.30	155.00	0.00
PC2	8.6	91.1	19	499.42	26.28	17.92	0.00
PC3	4.2	95.3	17	242.85	14.28	9.74	0.00
PC4	2.7	98.0	15	156.15	10.41	7.10	0.00
PC5	1.4	99.4	13	78.90	6.06	4.14	0.00
PC6	0.6	100.0	11	36.75	3.34	2.28	0.01

**Table 5:** Mean monthly temperatures, wind speed, and rainfall data during the field experiment period in Satkhira and Khulna.

Satkhira					Khulna			
Month	Maxi.	Min.	Wind (Km/h)	Rainfall	Maxi.	Min.	Wind (Km/h)	Rainfall
January 2018	27.50	17.25	8.0	0	27.5	18.25	8.7	0
January 2019	29.75	18.75	9.0	0	29.5	19.00	8.2	0
February 2018	33.25	22.25	7.0	0	32.75	21.5	7.5	0
February 2019	33.00	21.75	7.8	3.42	32.5	21.75	7.3	5.85
March 2018	37.75	25.75	9.5	0	36.5	25.25	8.5	0.3
March 2019	35.50	24.75	12.8	0.25	34.75	24.00	10.8	0.70
April 2018	35.50	27	14.5	4.97	34.25	26.75	12.3	10.6
April 2019	38.75	27.5	19.5	0.62	37.25	27.00	14.5	2.1

## Data Availability

Not applicable.
